# Mad Honey Ingestion Leading to Grayanotoxin Poisoning During the Burning Man Music Festival: A Case Series

**DOI:** 10.7759/cureus.62755

**Published:** 2024-06-20

**Authors:** Humaira Ali, Courtney Chiu, Nathan Woltman, Matt Samuel Friedman, Elie Harmouche

**Affiliations:** 1 Emergency Medicine, Maimonides Medical Center, Brooklyn, USA; 2 Emergency Medicine, Cleveland Clinic Foundation, Cleveland, USA; 3 Medical Toxicology, Maimonides Medical Center, Brooklyn, USA

**Keywords:** remote wilderness, grayanotoxin, prehospital ems, substance recreational use, toxicology and envenomation

## Abstract

This is a case series of three patients who presented to the medical facilities at Burning Man, an annual week-long gathering in the Black Rock Desert of Nevada, for recreational grayanotoxin ingestion. Grayanotoxin, also known as "mad honey," caused the patients to present with varying degrees of dizziness, nausea, vomiting, and diarrhea based on the quantity ingested. Vital signs showed significant bradycardia and hypotension and were successfully treated with atropine and intravenous fluids. Patients were later discharged after a period of observation and resolution of symptoms.

## Introduction

"Mad honey" refers to a type of honey that is produced by bees in some regions, particularly in Nepal, Turkey, and the Black Sea region, that collect nectar from specific rhododendron and azalea flowers, and other related plants containing grayanotoxins [1]. Mad honey is typically used for its purported medicinal benefits and recreationally for its psychoactive properties [1]. Mad honey intoxication is becoming an increasingly important concern due to increased availability and consumption of imported or locally produced honey. 

We report a case series of multiple casualties from mad honey ingestions who presented with symptoms consistent with grayanotoxin poisoning. Three patients presented with chief complaints of vomiting and diarrhea. The individuals report drinking an undisclosed amount of bourbon and mad honey, with the intention of creating natural hallucinogenic effects similar to ayahuasca.

## Case presentation

Methods

This is a case series that describes and analyzes the clinical presentations and outcomes of three individuals who ingested mad honey during Burning Man, a week-long communal event in the Black Rock Desert, approximately 115 miles north of Reno, Nevada. Three patients presented simultaneously to the acute care emergency department in the Black Rock Desert over a span of 30 minutes following ingestion of mad honey. Informed consent for participation in the case series was obtained from each patient. Multiple data points were collected through a review of electronic medical records and interviews of the treating physicians. Information regarding demographics, medical history, symptoms, physical examination findings, diagnostic procedures, treatments administered, and outcomes were systematically extracted.

Case 1

**Figure 1:**
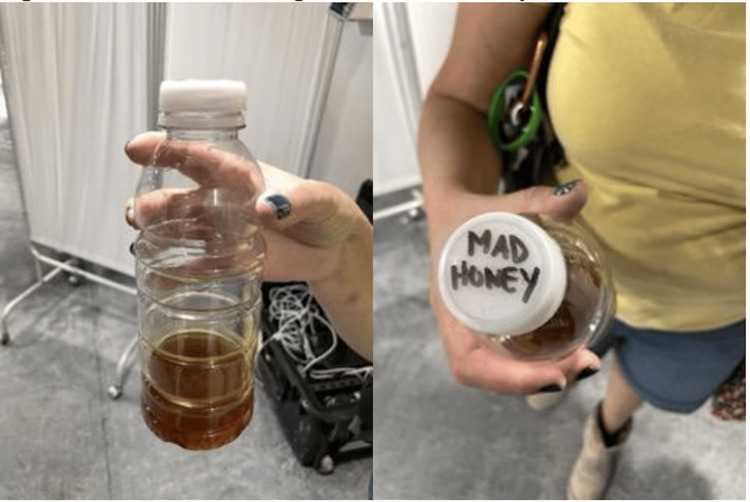
Remaining mixture of mad honey and bourbon

A 31-year-old male presented to the medical tent after ingesting an undisclosed amount of mad honey mixed with bourbon containing 43% alcohol by volume (Figure 1). He initially described a tingling sensation in the throat post-ingestion, which quickly resolved. He complained of multiple episodes of cyclical vomiting that began 45 minutes after ingestion followed by multiple episodes of non-bloody diarrhea. He denied abdominal pain but reported feeling warm and restless. Patient denied any other ingestions, prior medical problems, or current medication use.

His initial vital signs showed a blood pressure (BP) of 80/50 mmHg, heart rate (HR) of 44 beats per minute (bpm) and oxygen saturation of 100%. His physical exam was significant for pupils 6 mm bilaterally and reactive, clear lungs, and positive bowel sounds. No clonus, hyperreflexia or fasciculations were noted.

**Figure 2:**
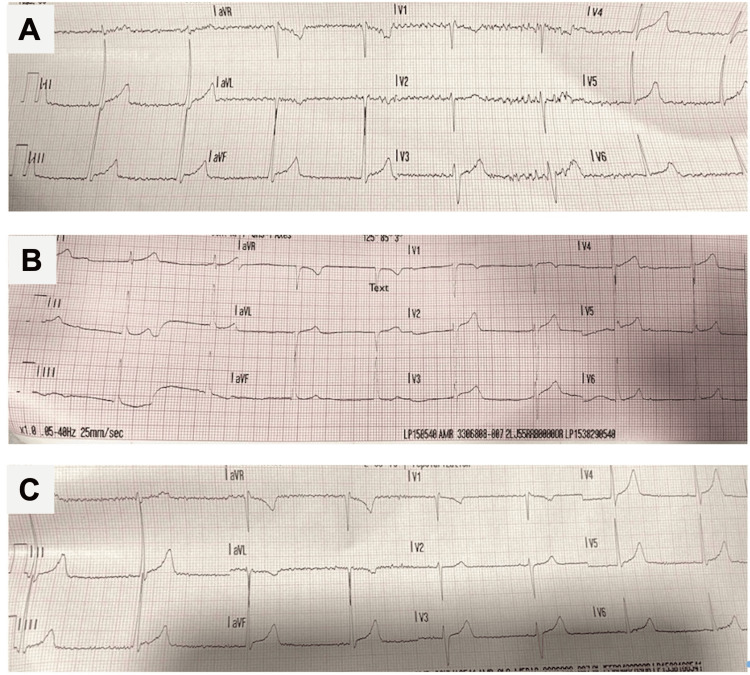
Initial presenting 12-lead electrocardiogram (EKG) for A: patient #1, B: patient #2, and C: patient #3

Patient was initially treated with two doses of ondansetron 4 mg intravenous (IV) 20 minutes apart. He continued to vomit and received metoclopramide 10 mg IV and two liters of 0.9% normal saline. Despite initial treatment, the patient remained hypotensive and continued to vomit. Given the patient's hypotension and bradycardia, he was given atropine 0.5 mg IV, which improved his HR to 75 bpm and his BP to 110/73 mmHg. The electrocardiogram (EKG) obtained after administration of atropine showed normal sinus rhythm (NSR) with a heart rate of 75 bpm and a normal axis (Figure 2). The patient stated that his nausea resolved after initial treatment, but loose stools persisted. At this time, the patient stated that he felt well and ambulated to the restroom without evidence of orthostasis or changes in vital signs.

Three hours after the initial presentation, the patient asked to ambulate to the bathroom, but upon return was pale and tired appearing. His vitals were repeated, and he was found to have stable blood pressure, but noted to be bradycardic to mid-50s. He received an additional liter of intravenous fluids

Point of care laboratory results are reported in Table 1. The patient was observed and discharged eight hours after presentation. Upon discharge, he reported feeling sleepy but denied alterations in mental status or perceptual abilities.

**Table 1:** Presenting point of care laboratory results (Analyzer: Piccolo Xpress, Abaxis, Union City, CA, USA)

	Patient #1	Patient #2	Patient #3	Normal Ranges
Sodium	141	147	143	[135-149 MMOL/L]
Potassium	3.7	5.5	3.9	[3.4-4.8 MMOL/L]
Bicarbonate	26	22	24	[22-32 MMOL/L]
Chloride	110	114	108	[93-105 MMOL/L]
Glucose	105	85	87	[59-140 mg/dL]
Calcium	10.1	10.4	10.1	[8.2-10.1 mg/dL]
Blood urea Nitrogen	15	13	15	[7-21 mg/dL]
Creatinine	1.5	1.9	1.7	[0.3-1.1 mg/dL]
Alkaline Phosphatase	56	40	92	[36-112 IU/L]
Alanine Transaminase	25	70	16	[6-47 IU/L]
Aspartate transaminase	31	77	29	[10-33 IU/L]
Total Bilirubin	0.6	0.6	0.3	[0.2-1.4 mg/dL]
Albumin	3.9	4.3	3.9	[3.5-5.2 g/dL]
Protein	7.2	7.2	7.5	[5.6-7.6 g/dL]

Case 2

A 38-year-old male with no past medical history presented with vomiting and diarrhea after ingesting three shots of mad honey mixed with bourbon one hour prior to presentation. Patient reported taking an undisclosed amount of ketamine prior to the ingestion. He stated that he felt a numb sensation in his throat after ingestion that rapidly resolved. He complained of multiple episodes of non-bilious, non-bloody emesis as well as multiple episodes of diarrhea associated with incontinence that started one hour prior to presentation. He reported a warm sensation in his extremities described as pins and needles associated with restlessness.

Initial vitals showed a heart rate of 50 bpm, blood pressure 84/45, and oxygen saturation of 99%. Physical exam revealed clear lungs, active bowel sounds and an intact neurologic status with bilateral reactive pupils measuring 5 mm. There was no evidence of clonus, hyperreflexia or fasciculations. Initial EKG demonstrated sinus bradycardia with a heart rate of 51 without evidence of prolonged QRS or QT intervals (Figure 2).

The patient received a total of ondansetron 8 mg IV and 2 L IV fluids in the first hour of presentation with no change in vital signs. The patient was subsequently given atropine 0.5 mg IV. His heart rate improved to 113 bpm, blood pressure increased to 114/62 mmHg, pupils were 3 mm and reactive. The patient reported his nausea improved, and he had no further bowel movements. However, symptoms return within two hours so additional intravenous fluid were administered.

Laboratory values are listed in Table 1. The patient was observed for six hours and ultimately discharged. Lowest heart rate during the observation period was 60 bpm without associated hypotension. Patient endorsed drowsiness without associated confusion or alteration in perception at any point after the ingestion.

Case 3

A 35-year-old male presented with vomiting and increased flatus after drinking three shots of mad honey and bourbon one hour prior to presentation. He admitted to ingesting an unknown amount of alcohol, but denied any other substances. The patient endorsed multiple episodes of non-bilious non-bloody vomitus that started immediately after consumption. He denied having loose stool but reported an increased amount of flatus.

Initial vitals revealed a heart rate of 50 bpm, systolic blood pressure of 84, and oxygen saturation of 99%. Physical exam again revealed clear lungs, active bowel sounds and an intact neurologic status with bilateral reactive pupils measuring 4 mm. There was no evidence of clonus, hyperreflexia or fasciculations and the patient was able to ambulate without ataxia. Initial EKG showed sinus bradycardia, no ST segment changes, no QRS widening, and no QTc prolongation (Figure 2).

The patient receives a total of ondansetron 8 mg IV and 2 L IV fluids. Repeat vitals are unchanged from presentation. He then receives atropine 0.5 mg IV. His heart rate increased to 115 and blood pressure increased to 110/54. Patient states that the nausea improved and did not have any bowel movements. Pupils are 3 mm and reactive. His symptoms recrudesced two hours after atropine administration as noted with bradycardia without hypotension and he is treated conservatively with IV fluids. Laboratory values are listed in Table 1.

Patient was observed for six hours. He ambulated to the bathroom but returned with bradycardia without hypotension and endorsed drowsiness. As with the previous patient in case 2, this patient also denied any altered sensorium.

## Discussion

Mad honey has been used in traditional medicine for various purposes. It has been claimed to have therapeutic effects for conditions such as hypertension, diabetes, gastrointestinal complaints, such as gastritis and peptic ulcers, arthritis and sexual dysfunction. The scientific evidence supporting these claims is limited [1,2].

Consuming excessive amounts of mad honey can lead to poisoning secondary to grayanotoxins. Some individuals seek out mad honey for its potential psychoactive effects which include, hallucinations, euphoria, and a feeling of intoxication [1]. However, the effects can be unpredictable and can regularly be accompanied by nausea and dizziness. Serious reported adverse effects include alterations in consciousness, seizures, and many cardiovascular events such as hypotension, atrial-ventricular blocks, atrial fibrillation and other cardiac dysrhythmias including asystolic arrest. Additionally, syncope, diplopia, and salivation have been reported. Symptoms mostly commonly occur within 20 minutes to three hours after ingestion and last for as long as one to two days. The metabolism and excretion rates can vary among individuals. Research on these toxins and their pharmacokinetics is limited [3,4].

Grayanotoxins are a group of diterpenoids found in several plant species of the Ericacea family. They primarily affect the voltage-gated sodium channels in cell membranes, leading to an increase in sodium ion influx and subsequent depolarization of the affected cells. This effect is particularly pronounced in cardiac myocytes and neuronal synapses due to the sensitivity of those sodium channels and subsequent prolonged activation from depolarization. Grayanotoxin binds to the sodium channel modifying its configuration thereby leaving the cell in a state of depolarization or activation [3]. Symptoms are caused by an inability to deactivate neural sodium ion channels resulting in continuous increased vagal tone [2,5].

Determining a safe or appropriate dose of mad honey is challenging due to several factors including concentrations of grayanotoxins in different honey samples, individual sensitivity, and potential for and variability in adverse effects. The concentration of grayanotoxins can vary depending on the specific plant sources and geographical locations. A well-defined therapeutic or medicinal dose of mad honey has not been established due to the wide range of toxic variability from person to person [2].

This case series is consistent with prior reported grayanotoxin toxicity. Hypotension, sinus bradycardia, dizziness, nausea and vomiting are most common presenting signs and symptoms [4]. Apart from these side effects, shock, acute myocardial infarction, Wolff-Parkinson-White syndrome, and complete heart block may occur [6-8]. The majority of patients are managed successfully with atropine in varying doses (0.5mg-1mg) and intravenous fluids and discharged within 24 hours [3,4,9].

Limitations

Grayanotoxin poisoning in the reported patients was confirmed clinically using the reported history, multiple similar presentations and response to treatment. The Naranjo Scale is a questionnaire designed by Naranjo et al. for determining the likelihood of whether an adverse drug reaction is actually due to the drug rather than the result of other factors. The timeline and presentation are consistent with grayanotoxin poisoning with a Naranjo scale of 9 consistent with a definite adverse drug reaction [10]. Of note, two additional patients took mad honey and did not present to the ED. There is no laboratory confirmation of grayanotoxin from biological specimens or residual ingested material and we are unable to rule out coingestants.

## Conclusions

Grayanotoxin poisoning is likely to remain an uncommon source of poisoning. However it may become increasingly prevalent as imported mad honey is unregulated and therefore more accessible to consumers who are looking for natural alternatives as medicines and/or mind-altering substances. Early recognition based on exposure and symptoms is important as a treatable toxicological etiology, especially as confirmatory studies are often not available.
